# A Risk Model Developed Based on Necroptosis Predicts Overall Survival for Hepatocellular Carcinoma and Identification of Possible Therapeutic Drugs

**DOI:** 10.3389/fimmu.2022.870264

**Published:** 2022-03-29

**Authors:** Zedong Li, Jianyu Fang, Sheng Chen, Hao Liu, Jun Zhou, Jiangsheng Huang, Sushun Liu, Yu Peng

**Affiliations:** ^1^ Department of General Surgery, The Second Xiangya Hospital, Central South University, Changsha, China; ^2^ Department of Gastrointestinal Surgery, West China Hospital, Sichuan University, Chengdu, China; ^3^ Clinical Nursing Teaching and Research Section, The Second Xiangya Hospital, Central South University, Changsha, China; ^4^ Department of Emergency Medicine, The Second Xiangya Hospital, Central South University, Changsha, China

**Keywords:** necroptosis, hepatocellular carcinoma, gemcitabine, TOP2A, regulated cell death

## Abstract

**Background:**

Necroptosis is a form of regulatory cell death (RCD) that attracts and activates immune cells, resulting in pro-tumor or anti-tumor effects. The purpose of this study was to investigate genes associated with necroptosis, to construct a risk score for predicting overall survival in patients with hepatocellular carcinoma, and to find potentially effective drugs.

**Methods:**

The three algorithms ssGSEA, EPIC, and ESTIMATE were used to quantify the immune cell infiltration of the samples, differentially expressed genes (DEGs) analysis, and weighted gene co-expression network analysis were used to screen necroptosis related genes. Variables were screened according to random survival forest analysis, and combinations with significant p-values and a low number of genes were defined as prognostic signatures by using log-rank test after gene combination. Based on the sensitivity data of PRISM and CTRP2.0 datasets, we predicted the potential therapeutic agents for high-NRS patients.

**Results:**

Seven genes such as TOP2A were used to define necroptosis-related risk score (NRS). The prognostic value of risk score was further validated, where high NRS was identified as a poor prognostic factor and tended to have higher grades of histologic grade, pathologic stage, T stage, BCLC, CLIP, and higher AFP. Higher NRS was also negatively correlated with the abundance of DCs, Neutrophils, Th17 cells, Macrophages, Endothelial, and positively correlated with Th2 cells. Necroptosis is often accompanied by the release of multiple cytokines, and we found that some cytokines were significantly correlated with both NRS and immune cells, suggesting that necroptosis may affect the infiltration of immune cells through cytokines. In addition, we found that TP53 mutations were more common in samples with high NRS, and these mutations may be associated with changes in NRS. Patients with high NRS may be more sensitive to gemcitabine, and gemcitabine may be an effective drug to improve the prognosis of patients with high NRS, which may play a role by inhibiting the expression of TOP2A.

**Conclusions:**

We constructed a necroptosis-related scoring model to predict OS in HCC patients, and NRS was associated with immune response, TP53 mutation, and poor clinical classification in HCC patients. In addition, gemcitabine may be an effective drug for high-NRS patients.

## Introduction

Hepatocellular carcinoma (HCC) is one of the most common solid malignancies and is the leading cause of cancer-related deaths worldwide (1). Despite new advances in the molecular basis and new chemotherapeutic approaches, only a slight decrease in mortality has been achieved (2). Tumorigenesis in HCC is a complex process involving multiple risk factors.

Chronic hepatitis B (HBV) and hepatitis C virus (HCV) infection, alcohol consumption, nonalcoholic fatty liver disease (NAFLD), and aflatoxin B1 all contribute to the development of HCC (3). Although there are several treatment options available for HCC, overall survival is still far from satisfactory.

Regulated cell death (RCD), a fundamental biological phenomenon of cells, has an irreplaceable impact on the onset and development of many processes of the disease. RCD includes apoptosis, autophagy, necroptosis, pyroptosis, paraptosis, and neutrophils NETosis (4).

Resistance to RCD, especially apoptosis, is one of the major hallmarks of cancer and has long been a major obstacle to anticancer therapy (5, 6). Studies have shown that the expression levels of RIPK3 and MLKL correlate with the susceptibility to necroptosis, and caspase-8 is the most critical factor in the prevention of necroptosis (7). RIPK3-mediated epithelial necroptosis can lead to intestinal inflammation (8), and the lack of RIPK3 was shown to prevent skin inflammation in mice (9). Inflammation itself can also regulate necroptosis through cytokines such as type I/II IFN (10). Necroptosis has been shown to trigger inflammation (11), and some investigators believe that necroptosis is a more potent inducer of inflammation than apoptosis, but rigorous experimental validation is lacking (7). In addition, necroptosis may be immunogenic and even exhibit identifiable immunogenic cell death-like features (12).

The role of RCD in tumors seems to be a double-edged sword. Liver aging is associated with increased necroptosis, which leads to chronic inflammation of the liver, and chronic inflammation of the liver appears to lead to liver fibrosis and possibly chronic liver disease (13). Primary hepatocellular carcinoma often occurs in chronically damaged livers where different types of cell death occur, such as necrosis, apoptosis, or necroptosis. When necroptosis predominates in the liver microenvironment, hepatocytes with abnormally activated oncogenes lead to cholangiocarcinoma. However, hepatocytes with the same oncogenic drivers will cause HCC if they are not adjacent to necroptosis-dying hepatocytes (14). Although studies have revealed the important role of necroptosis in numerous cancers, even in hepatocellular carcinoma, the exact mechanism remains unknown.

In this study, sequencing data and clinical information of HCC patients were downloaded from public databases. Then, differentially expressed genes (DEGs) analysis, weighted gene co-expression network analysis, and random survival forest analysis were used to screen necroptosis-related prognostic genes. Based on these selected genes, necroptotic-related risk score (NRS) was calculated by multivariate COX analysis. In addition, we found that patients with high NRS may more sensitive to gemcitabine.

## Materials and Methods

### Data Source and Necroptotic Score

FKPM, counts, and clinical information of the TCGA-LIHC were downloaded from the UCSC Xena (https://xenabrowser.net/datapages/). In this work, 316 primary hepatocellular carcinoma samples with R0 resection from the TCGA-LIHC were included. The GSE14520 dataset contains 220 non-tumor tissue of HCC patients and 221 tumor tissue. The normalized expression profiles and clinical information were downloaded from the NCBI Gene Expression Omnibus (GEO, https://www.ncbi.nlm.nih.gov/gds/). Expression profiles and clinical information of LIRI-JP were downloaded from the International Cancer Genome Consortium (ICGC, https://dcc.icgc.org/), which includes a total of 240 samples for primary hepatocellular carcinoma. Biomarkers for gobp necroptotic signaling pathway and hallmark pathway were downloaded from the Molecular Signatures Database (https://www.gsea-msigdb.org/gsea/msigdb). Based on the “GSVA” R package (15), a single sample gene set enrichment analysis (ssGSEA) was used to calculate the enrichment score, which can be defined as necroptotic score for gobp necroptotic signaling pathway.

### Quantification of Immune Cell Infiltration

Firstly, according to the work of Bindea et al. (16), and based on ssGSEA, infiltration of 24 immune cells was identified. B cells, cancer-associated fibroblasts (CAFs), CD4^+^ T cells, CD8^+^ T cells, endothelial cells, macrophages and NK cells, the infiltration of 8 immune cells were analyzed by EPIC (17). The composition and abundance of immune cells in mixed cells can be estimated by CIBERSORT (18). The algorithms CIBERSORT and EPIC were included in the IOBR R package (19), and finally, the IOBR R package was used to quantify immune cells.

### Kaplan–Meier Analysis and Differentially Expressed Genes Analysis

The Survminer R package performed to screen the best cut-off point for all continuous variables in the current work. Based on the grouping of best cut-off point, DEGs analysis was further carried out. Volcano maps and Kaplan–Meier curve were drawn by Ggplot2 and survival R packages, respectively.

### Weighted Gene Co-Expression Network Analysis (WGCNA) and Development of Necroptotic-Related Risk Score

WGCNA was applied to identify highly coordinated gene modules. The genes with variances, greater than the quartiles of all variances for WGCNA, were selected to identify gene modules, which are closely related to the necroptosis scores. Modules with |correlation coefficient of |above 0.4 were considered as necroptosis-correlated modules.

DEGs of the TCGA-LIHC and GSE14520 datasets and genes in necroptosis-correlated modules were considered as necroptosis related genes that may affect OS or RFS. First of all, the TCGA-LIHC was used as a training set, where the intersection of the three sets of genes was taken, then the initial screen for genes associated with OS/RFS (P-value <0.05) was analyzed by univariate Cox regression. Eventually, the random forest was further downscaled to select the top ten genes of variable importance. The combined genes with significant p-values and a small number of genes were defined as the prognostic signatures by log-rank test. Prognostic signatures and multivariate COX regression were used to determine the coefficient for the final model. The GSE14520 datasets and LIRI-JP were used as validation sets, where the predictive accuracy of the NRS was evaluated by using time-dependent ROC. NRS can be divided into high and low groups according to the best cut-off point. Kaplan–Meier analysis was used to compare the prognostic differences between high- and low-NRS, and the hazard of the two groups was presented by Kernel-smoothing hazard function plot.

### Relationship Between Necroptotic-Related Risk Score and Immune Signatures

The relationship between NRS and immune signatures was evaluated based on two aspects (1): the expression of cytokine, the human leukocyte antigen (HLA) gene family and immune checkpoints (20–23), and (2) infiltration of immune cells that were calculated by EPIC, CIBERSORT, and ssGSEA. Pearson correlation was used to analyze the correlation between NRS and immune signatures, and the differences of gene expression and immune cells between High-NRS and low-NRS were compared by Wilcox test. Gene expression and infiltration of immune cells were displayed as Heatmap.

### Survival Analysis for Immune Cells

In the TCGA-LIHC and GSE14520 datasets, immune cells with a |correlation coefficient| >0.35 and significant group differences were included in the survival analysis, grouping by the best cut-off point. Immune cells with a P-value <0.05 were considered to have prognostic value. In addition, the correlation between immune cells and prognostic signatures was further calculated.

According to the marker of the hallmark pathway, gene set variation analysis was used to assess hallmark pathways, and then the difference analysis was performed by the limma R package to find pathways, which is significantly differed between the low-and high-NRS groups. The Fisher’s test was used to compare the differences in clinical phenotypes between the two groups.

### Mutation Status in the Low- and High-NRS

We downloaded mutation data from the UCSC Xena, extracted genes with mutation rates greater than 10% for permutation test, and retained genes with P-value <0.05. Then, the TCGAbiolinks R package was used to download simple nucleotide variation data of the TCGA-LIHC, and a lollipop chart was used to display mutation sites of TP53 proteins.

### Prediction of Potential Therapeutic Agents in Patients With High-NRS

Based on the sensitivity data of PRISM (https://www.theprismlab.org/) and CTRP2.0 (https://portals.broadinstitute.org/ctrp.v2.1/) and “pRRophetic” R package, we predicted the potential therapeutic agent for high-NRS patients. First, top and bottom deciles of NRS were selected for differential drug response analysis, and compounds with log2FC >0.2 were identified as potential therapeutic agents which estimated AUC values were lower. Spearman correlation analysis were used to identify the correlation between compounds AUC value and NRS, indicating that compounds with correlation coefficient <−0.3 imply a smaller AUC value in high-NRS.

The GSE116444 dataset contains genes expression of NCI-60 cell lines before and after exposure to gemcitabine. GDSC (https://www.cancerrxgene.org/) and CCLE (https://portals.broadinstitute.org/ccle) provide tumor cell line drug sensitivity information and corresponding genomics information. The potential therapeutic agents can be further verified by the GSE116444 dataset, GDSC, and CCLE.

## Results

### Calculation of Necroptosis Scores and Kaplan–Meier Analysis

**Figure 1:**
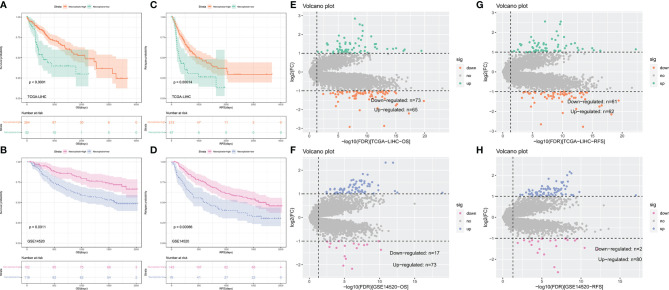
Kaplan–Meier curves comparing the high and low necroptosis score and differentially expressed genes (DEGs) analysis. **(A–D)** Kaplan–Meier curves of OS and RFS in TCGA-LIHC and GSE14520. **(E–H)** Volvano plot of DEGs analysis in TCGA-LIHC and GSE14520.

The flow chart of this study is shown in **Supplementary Figure 1**. A total of 316 TCGA-LIHC patients and 221 patients of GSE14520 (221 tumor tissues) were included in this study to calculate necroptosis scores. A summary of clinical characteristics can be found in **Supplementary Table 1**. Different necroptosis scores show different prognoses, where a higher necroptosis score is associated with a worse prognosis (**Figures 1A, B**) and earlier relapse (**Figures 1C, D**). This result was found in both the TCGA-LIHC dataset and the GSE14520 dataset.

**Figure 2:**
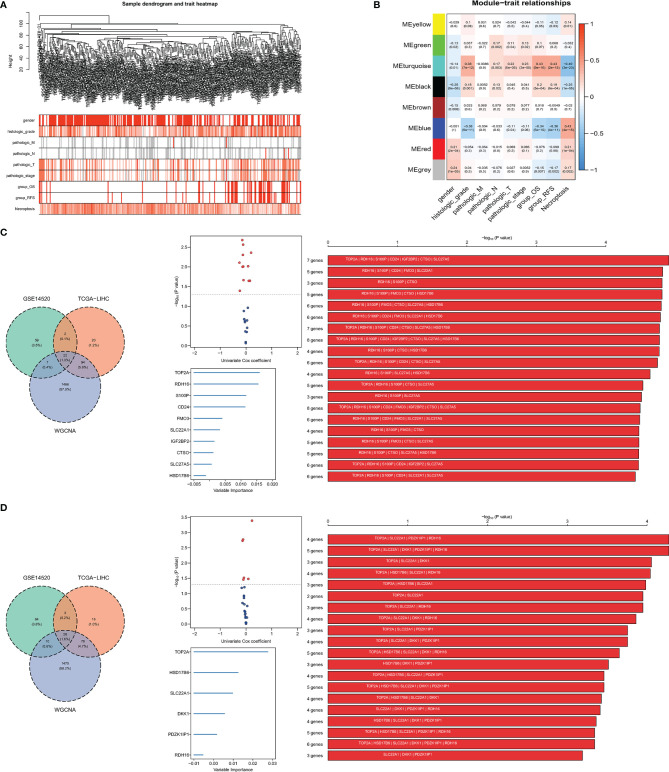
Screening for necroptosis-related genes. **(A)** Clustering dendrogram. **(B)** Correlation between gene modules and necroptosis scores; **(C, D)** signature combination for predicting OS and RFS, the intersection of DEGs and selected module genes, volcano plot showed the genes of the intersection of the univariate Cox regression analysis, signature combination selected by random survival forest analysis and Kaplan–Meier analysis.

To identify hub genes for necroptosis in hepatocellular carcinoma and construct a risk score, we performed genes difference analysis and WGCNA. Grouping for gene difference analysis were based on groups with different OS and RFS, and DEGs with p-value <0.05 and |log fold change| (logFC) >1 were selected. In DEGs of OS, 73 downregulated and 65 upregulated genes of the TCGA-LIHC dataset (**Figure 1E**) and 17 downregulated and 73 upregulated genes of the GSE14520 dataset (**Figure 1F**) were included. In DEGs of RFS, 61 downregulated and 62 upregulated genes of the TCGA-LIHC dataset (**Figure 1G**) and 2 downregulated and 8 upregulated genes of the GSE14520 dataset (**Figure 1H**) were included.

### Weighted Gene Co-Expression Network Analysis

The variance of all genes in the TCGA-LIHC dataset and ¼ of all genes with the larger variance were selected and calculated, where a total of 3,382 genes were included in the WGCNA. After clustering, four outlier samples were removed and a total of 312 samples were included, and a sample clustering tree was drawn (**Figure 2A**). Finally, in addition to the gray module, seven gene modules were identified. The turquoise module was highly related to the histologic grade, OS and RFS, and negatively correlated with necroptosis score. The blue module was negatively correlated to histologic grade, OS, and RFS, and highly related to necroptosis score (**Figure 2B**). Thus, turquoise and blue modules were selected as important modules related to OS, RFS, and necroptosis for further analysis.

### Construction of Necroptotic-Related Risk Score

**Figure 3:**
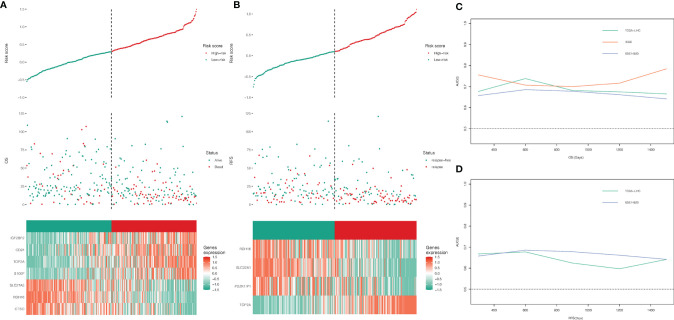
**(A, B)** Analysis of NRS in OS and RFS, grouped by median; **(C, D)** time-dependent ROC curves.

A total of 22 genes in both DEGs of the TCGA-LIHC and GSE14520 datasets and turquoise and blue modules, associated with necroptosis have both predictive values for OS. Twelve genes with P-value <0.05 were selected by univariate Cox regression analysis, and 1,023 combinations of top 10 genes with higher variable importance were sorted according to the P-value of KM. The top 20 signatures are shown in **Figure 2C**, where the signature included 7 genes (RDH16, S100P, TOP2A, CD24, IGF2BP2, CTSO and SLC27A5) with a big −log10 p-value and a small number of genes were selected. The risk score for OS was calculated by the formula: NRS = TOP2A ∗ 0.16898956 − RDH16 ∗ 0.16898956 + S100P ∗ 0.05429773 + CD24 ∗ 0.02580513 + IGF2BP2 ∗ 0.01426958 − CTSO ∗ 0.11553961 + SLC27A5 ∗ 0.08462284. Similarly, a 4-gene (TOP2A, SLC22A1, PDZK1IP1, RDH16) combination for predicting cancer recurrence was selected (**Figure 2D**). Notably, both TOP2A and RDH16 were included in the survival and relapse models. Then, risk prediction formulas for OS and RFS and risk scores were designed and calculated, respectively. The results show that among patients with high-risk scores, more patients died or relapsed (**Figures 3A, B**), where IGF2BP2, CD24, TOP2A, and S100P have higher gene expression in patients with a high-risk score of OS. Moreover, SLC27A5, RDH16, and CTSO have higher gene expression in patients with a low-risk score of OS (**Figure 3A**). RDH16, SLC22A1, and PDZK1IP1 have higher gene expression in patients with a low-risk score of RFS, and TOP2A have higher gene expression in patients with a high-risk score of RFS (**Figure 3B**).

### Evaluation and Validation for the Prognostic Value of the Risk Scores

**Figure 4:**
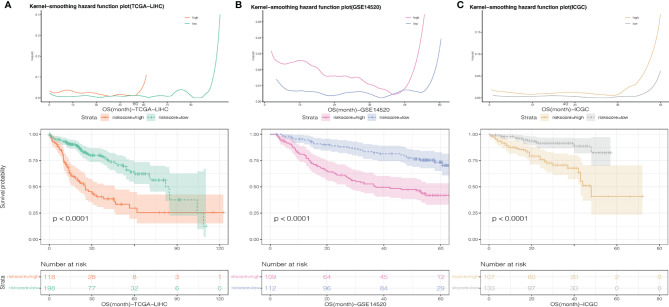
Smoothed hazard estimates of patients with low- and high-NRS according to survival analysis. **(A)** TCGA-LIHC, **(B)** GSE14520, **(C)** C:ICGC-LIHC.

To further evaluate the predictive value of risk scores, time-dependent ROC curves were used to assess the prognostic accuracy of the two risk scores on OS and RFS. The AUC in predicting overall survival was around 0.7 (**Figure 3C**), which is considered a high prognostic factor. However, the AUC in predicting relapse-free survival was lower than 0.7 (**Figure 3D**) and was considered a poor prognostic factor. Given that the risk score for OS has a good predictive effect, we defined the risk score based on 7 genes as the necroptosis-related risk score (NRS). The survival analysis showed that in the TCGA-LIHC, GSE14520, and ICGC data sets, the overall survival of patients with high-NRS was significantly lower than that of patients with low-NRS. The hazard curve grouped by NRS showed that the first peak of the risk of death in the high-NRS group appeared earlier than that in the low-NRS group. Moreover, the hazard of the high-NRS group is always higher than that of the low-NRS group (**Figure 4**).

**Figure 5:**
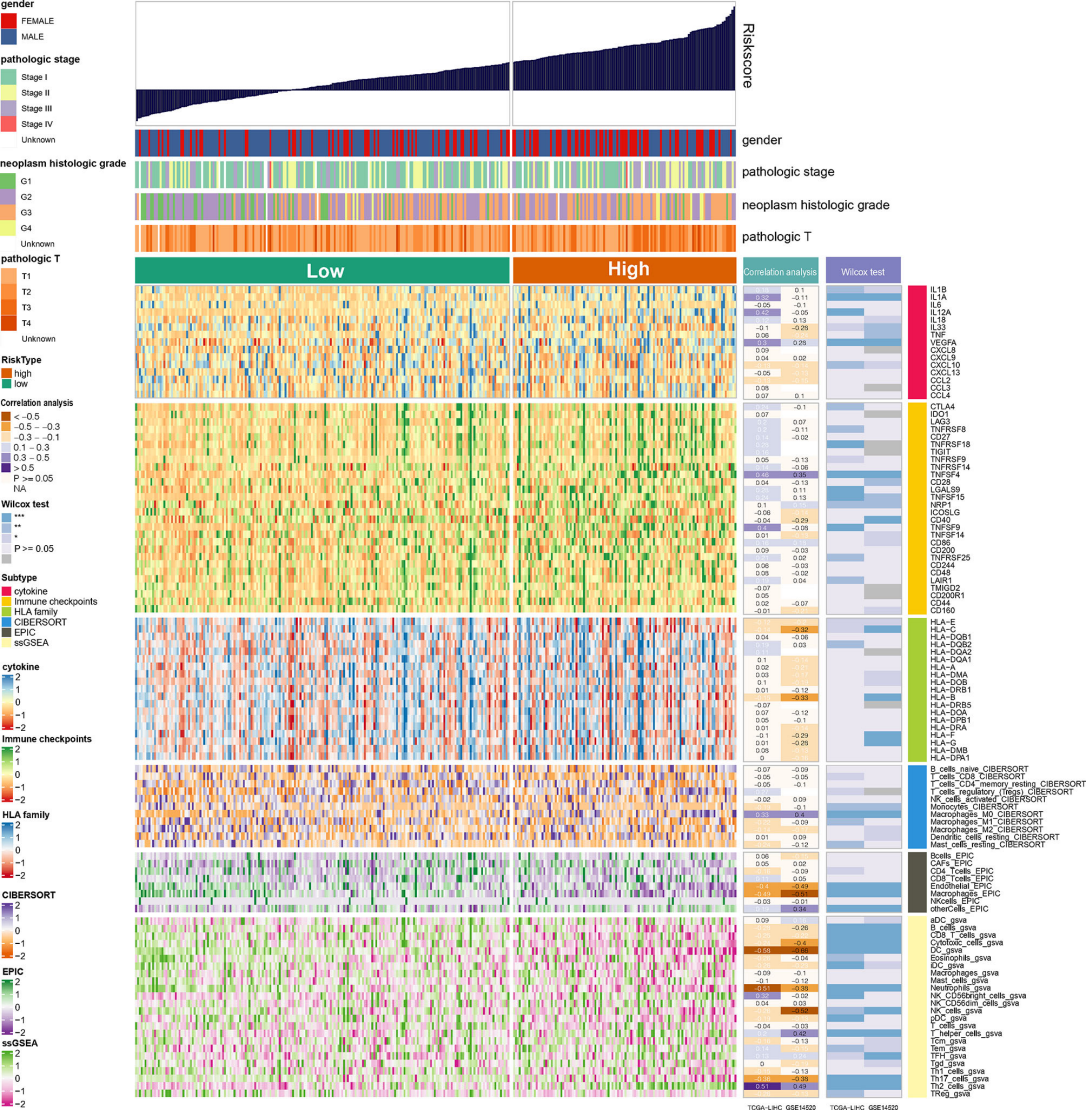
Landscape of cytokine, immune checkpoints, HLA family genes, and immune cell infiltrations in the low- and high-NRS groups. Correlation analysis for NRS and expression and immune infiltration. Wilcox tests were used to detect the difference in genes expression and immune infiltration between low- and high-NRS groups. *p<0.05, **p<0.01, ***p<0.001.

Wilcoxon test was used to compare the differences in cytokine, immune checkpoints, and HLA family genes expression between the high- and low-NRS groups. Pearson correlation analysis was applied to identify the correlation between NRS and these gene expressions. In the GSE14520 and TCGA-LIHC datasets, our results indicate that VEGFA of cytokine and TNFSF4 of immune checkpoints have shown significant differences in expression between the high- and low-NRS groups, and a significant positive correlation with NRS was obtained. Quantification of immune cell infiltration was carried out by CIBERSORT, EPIC, and ssGSEA, and the results of Pearson correlation analysis and Wilcoxon test show that endothelial, macrophages, DC, neutrophils, and Th17 cells are significantly different from the high- and low-NRS groups, which are negatively correlated with NRS, Th2 cells but positively correlated with NRS (**Figure 5**). The inclusion criteria were: P-value <0.05 and correlation coefficient >0.3. Notably, macrophage infiltration analyzed by EPIC showed a significant correlation, which is in contrast to ssGSEA (no correlation). This could be due to the differences in algorithms and markers.

### NRS was Associated With LIHC Immune Signature

**Figure 6:**
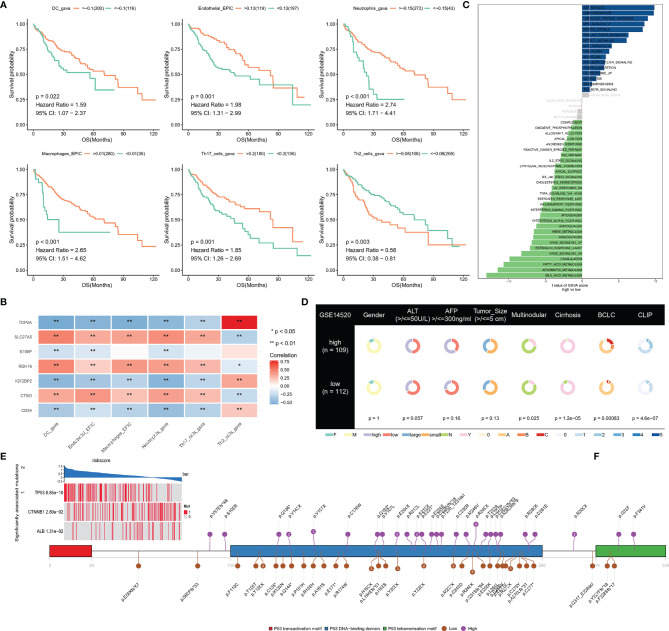
**(A)** Survival analysis of immune cells; **(B)** The correlation between signatures and immune cells; **(C, D)** Difference of hallmark pathways and clinical phenotypes between low- and high-NRS groups; **(E)** Mutant genes that are statistically different between low- and high-NRS groups; **(F)** mutation sites of TP53 in high- and low-NRS groups are independently summarized. *p<0.05, **p<0.01.

The prognostic value of immune cells and their relationship with NRS were then systemically researched. Survival analysis has shown that patients with high immune infiltration of endothelial, macrophages, DC, neutrophils, and Th17 cells have longer survival times than patients with high immune infiltration of Th2 cells (**Figure 6A**). In addition, correlation analysis has shown that TOP2A has a significant positive correlation with Th2 cells, and other hub genes also have a significant correlation with immune cell infiltration (**Figure 6B**). This finding implies that necroptosis may have synergistic or antagonistic effects with immune cells such as Th2 cells, Th17 cells, etc.

An analysis of hallmark pathway gene signatures revealed some differences of hallmark pathway between high- and low-NRS groups, E2F TARGETS, G2M CHECKPOINT, UNFOLDED PROTEIN RESPONSE, MYC TARGETS as the top enriched signatures in high-NRS groups, and FATTY ACID, XENOBIOTIC, and BILE ACID METABOLISM as the top enriched signatures in low-NRS groups (**Figure 6C**).

By comparing the clinical phenotypes between the two groups, we found that there were no statistical differences in gender, ALT, AFP, and tumor size in the GSE14520 dataset, which contains more clinical phenotype data, while multinodular, cirrhosis, Barcelona Clinic Liver Cancer (BCLC) and Cancer of the Liver Italian Program were statistically different (P-value <0.05). As the results show, more patients with cirrhosis and multiple nodules in the high-NRS group and the stage of BCLC and CLIP were higher in the high-NRS group (**Figure 6D**).

### Correlation Between Mutation and NRS (Independence)

To find significantly associated mutations, the differences in mutated genes between the two groups were compared by using a permutation test, which showed that TP53, CTNNB1, and ALB were significantly different. In particular, mutations in TP53 were commonly found in high-NRS patients (**Figure 6E**), where amino acid substitutions at p.R248 and p.P249 were observed in low-NRS patients and high-NRS patients respectively (**Figure 6F**).

### Gemcitabine as Potential Therapeutic Agent for High-NRS Patients

**Figure 7:**
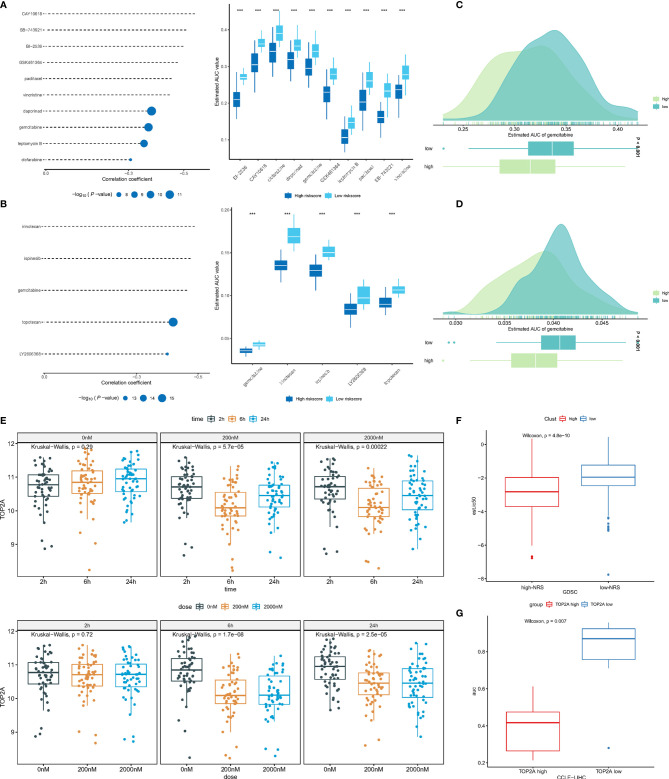
**(A)** Correlation and difference of CTRP-derived compounds; **(B)** Correlation and difference of PRISM-derived compounds; **(C)** AUC of gemcitabine (CTRP); **(D)** AUC of gemcitabine (PRISM); **(E)** The expression of TOP2A in different gemcitabine concentration and treatment time; **(F)** The difference of predicted IC50 between low- and high-NRS groups; **(G)** The difference of AUC between expression of TOP2A^high^ and TOP2A^low^ groups. ***p<0.001.

Compounds with a correlation coefficient < −0.3 and log2 FC >0.2 were identified as potential therapeutic agents, where eventually, ten CTRP-derived compounds (namely, BI-2536, CAY10618, clofarabine, daporinad, gemcitabine, GSK461364, leptomycin B, paclitaxel, SB-743921, and vincristine) (**Figure 7A**) and five PRISM-derived compounds (namely, gemcitabine, irinotecan, ispinesib, LY2606368, and topotecan) (**Figure 7B**) were identified. Notably, gemcitabine appears in CTRP-derived and PRISM-derived compounds. The estimated AUC of gemcitabine is shown in **Figures 7C, D**.

To further investigate the mechanism by which gemcitabine acts, the GSE116444 dataset was analyzed. The results showed that among the seven genes, only TOP2A exhibited expression changes after gemcitabine treatment (**Supplementary** **Figure 2**). After exposing to 200 and 2,000 nM gemcitabine, there was no significant change in TOP2A expression at 2, 6, and 24 h. The TOP2A expression showed a significant decrease (P-value <0.05). As mentioned earlier, the TOP2A expression was higher in the high-NRS group, suggesting that gemcitabine may reduce NRS and thus enhance patient prognosis by inhibiting TOP2A (**Figure 7E**). Based on the GDSC database, the IC50 of the TCGA-LIHC samples to gemcitabine was predicted by using the pRRophetic R package, where high-NRS samples have a lower IC50, which can be defined as statistically significant, suggesting that high-NRS has a stronger affinity for gemcitabine (**Figure 7F**). According to the median expression of TOP2A, the sample was divided into two groups; the results showed that the group of high TOP2A expression had lower drug sensitivity AUC (**Figure 7G**). In summary, gemcitabine might be a potential therapeutic agent to improve the prognosis of patients with high NRS.

## Discussion

In this study, we found the prognostic value of necroptosis in hepatocellular carcinoma and constructed an NRS system based on the necroptosis score. Our study showed that high-NRS had a worse prognosis, and immune infiltration of endothelial, macrophages, DC, neutrophils, and Th17 cells had a significant negative correlation with NRS and a significant positive correlation with Th2 cells. In addition, patients with high-NRS may be more sensitive to gemcitabine, and gemcitabine may be an effective drug to improve the prognosis of patients with high-NRS.

Many studies suggest that the various anti-tumor drugs currently in use may be linked to necroptosis (24). Further research into the role of necroptosis in tumors could help to use necroptosis to treat tumors. Necroptosis is a double-edged sword in the development of tumors (25), it can both promote (26) and inhibit (27, 28) tumor development. Liao et al. suggest that necroptosis inhibition may lead to sorafenib resistance in hepatocellular carcinoma (29). This result indicates the positive significance of necroptosis in the prognosis of HCC patients. Likewise, our findings suggest that patients with high necroptosis enrichment scores have a better prognosis, induction of necroptosis in hepatocellular carcinoma, and may help to improve the prognosis of HCC patients.

Necroptosis is associated with cytokines and pro-necroptosis proteins such as RIPK1 directly promote the production of cytokines such as IL6 (30), necroptotic cell death promotes autonomous cytokine production, and generates immune responses (31). Studies by Lomphithak et al. have shown that in cholangiocarcinoma, necroptosis is associated with a favorable immune cell signature (32). In addition, inflammation can also regulate necroptosis through cytokines such as IFN, increasing the expression of MLKL. Our study found that VEGFA of cytokine and TNFSF4 of immune checkpoints were significantly correlated with NRS and differed between high- and low-NRS groups. This suggests that cytokine and immune checkpoints may also be involved in necroptosis in gastric cancer.

Stimulation of cervical cancer cells with PolyIC induced necroptosis and led to activation of DC (33). This study showed that high NRS was negatively correlated with immune infiltration of endothelial cells, macrophages, DCs, neutrophils, and Th17 cells. Necroptosis driving pro-tumor/anti-tumor immune responses has been reported (12), and tumor cells have also been reported to induce necroptosis of endothelial cells, thereby promoting tumor cell extravasation and metastasis (26). It can be seen that necroptosis and its relationship with immune cells cannot be explained in a “black or white” way. Under different immune microenvironments, necroptosis and immune cells may have synergistic or antagonistic effects.

In addition to differences in the immune microenvironment between high- and low-NRS groups, we also found that high-NRS had higher TP53 mutations. In small−cell lung cancer, Sirtuin 3 induces necroptosis by regulating TP53 mutation (34), where higher TP53 mutations in gastric cancer may inhibit necroptosis and lead to a worse prognosis. As a risk factor for NRS score, TOP2A has also been reported to be associated with poor prognosis in pancreatic and colon cancers (35, 36). Dong et al. found that TOP2A may enhance HCC metastasis by mediating the p-ERK1/2/p-SMAD2/Snail pathway to promote EMT. In addition, many bioinformatic analysis results have found that TOP2A is a key gene for poor prognosis in HCC patients (37–39).

For the poor prognosis of high-NRS, we predicted potentially effective drugs, such as clofarabine, daporinad, and gemcitabine. Especially, gemcitabine may have a better effect in high-NRS patients and TOP2A may be the key gene for gemcitabine to play its role. Gemcitabine-induced necroptosis has been reported (40–42), which suggests that gemcitabine may be an effective drug in patients with high NRS by inducing necroptosis.

Although we constructed a necroptosis-related prediction model and identified potentially effective drugs, this study was based on data analysis, and we need to further validate the role of key genes and the effect of gemcitabine in future experiments.

## Conclusion

In conclusion, we constructed a necroptosis-related scoring model to predict OS in HCC patients, and NRS was associated with immune response, TP53 mutation, and poor clinical classification in HCC patients. In addition, gemcitabine may be an effective drug for high-NRS patients.

## Data Availability Statement

The datasets presented in this study can be found in online repositories. The names of the repository/repositories and accession number(s) can be found in the article/**Supplementary Material**.

## Author Contributions

ZL conceived the project and wrote the manuscript. ZL, JF, YP, HL and SC participated in data analysis. JZ and JF participated in discussion and language editing. JZ reviewed the manuscript. ZL, JH and SL revised the manuscript. All authors listed have made a substantial, direct, and intellectual contribution to the work and approved it for publication.

## Conflict of Interest

The authors declare that the research was conducted in the absence of any commercial or financial relationships that could be construed as a potential conflict of interest.

## Publisher’s Note

All claims expressed in this article are solely those of the authors and do not necessarily represent those of their affiliated organizations, or those of the publisher, the editors and the reviewers. Any product that may be evaluated in this article, or claim that may be made by its manufacturer, is not guaranteed or endorsed by the publisher.

## Supplementary Material

Supplementary Figure 1 The flowchart of the study procedures.

Supplementary Figure 2 Expression changes of 7 genes after gemcitabine treatment.

Supplementary Table 1 Summarizes of clinical characteristics.

## Abbreviations

DEGs, Differentially expressed genes; NRS, Necroptotic-related risk score; OS, Overall Survival; RFS, Relapse-free survival; ssGSEA, Single sample gene set enrichment analysis; WGCNA, Weighted correlation network analysis.
