# Supplementary figures and images for: A Risk Model Developed Based on Necroptosis Predicts Overall Survival for Hepatocellular Carcinoma and Identification of Possible Therapeutic Drugs
Source: Front Immunol. 2022 Mar 29;13:870264. doi: 10.3389/fimmu.2022.870264 (PMC9001936; doi:10.3389/fimmu.2022.870264)

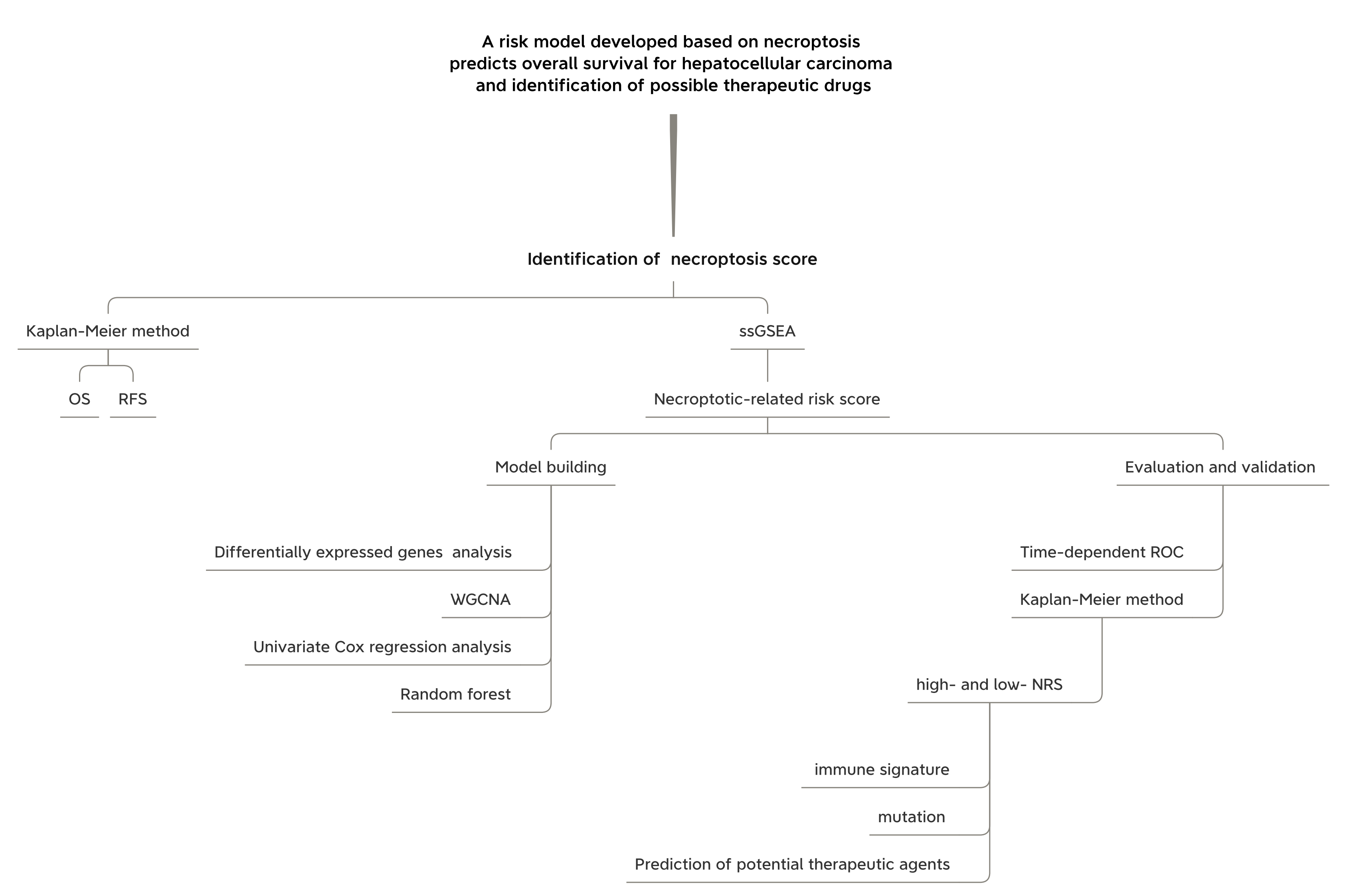

Supplement: Supplementary Figure 1 — The flowchart of the study procedures. [file Image_1.tif]

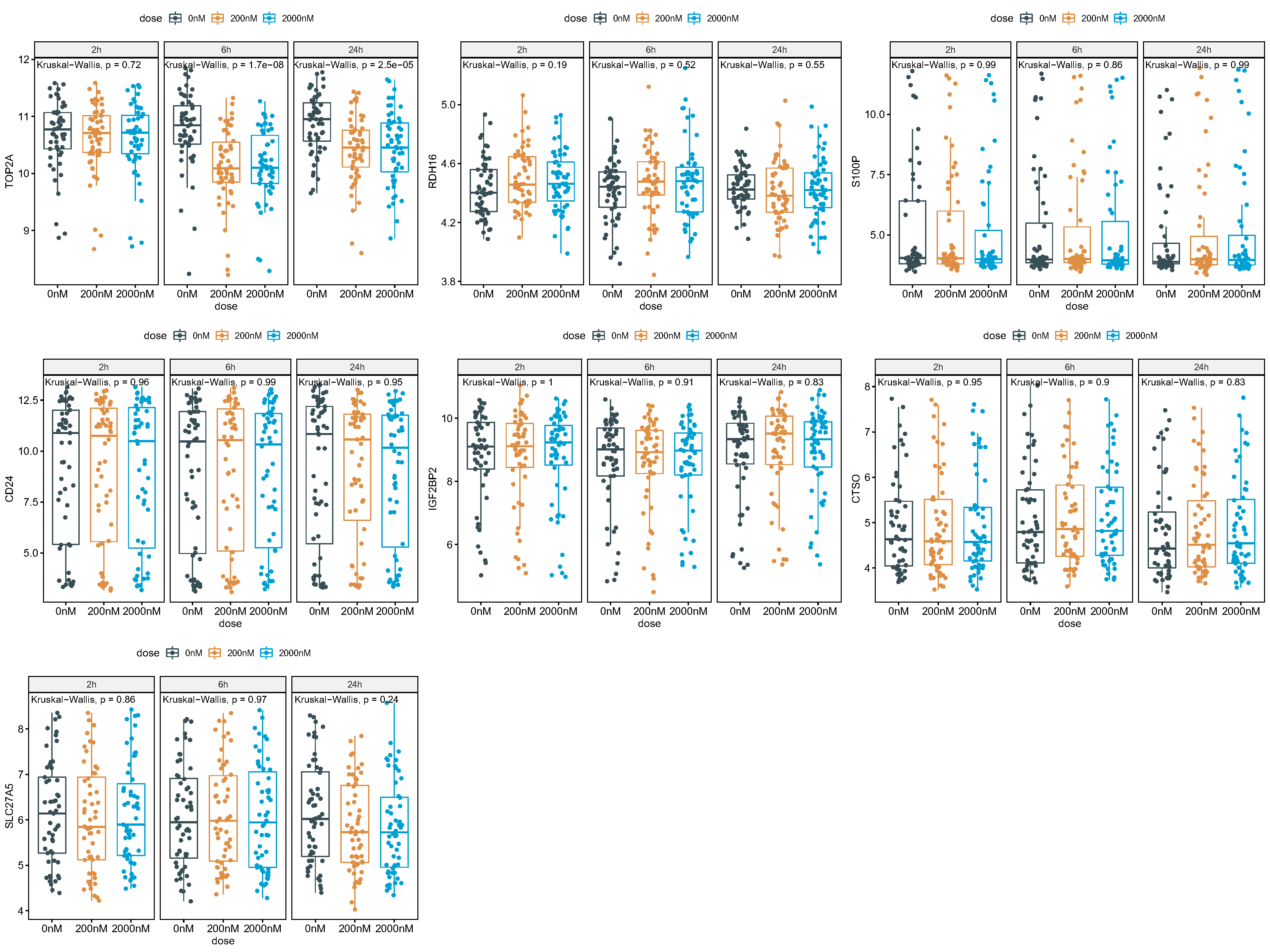

Supplement: Supplementary Figure 2 — Expression changes of 7 genes after gemcitabine treatment. [file Image_2.tiff]
